# Kidney Organoids: Current Advances and Applications

**DOI:** 10.3390/life15111680

**Published:** 2025-10-29

**Authors:** Hiroyuki Nakanoh, Kenji Tsuji, Kazuhiko Fukushima, Naruhiko Uchida, Soichiro Haraguchi, Shinji Kitamura, Jun Wada

**Affiliations:** 1 Department of Nephrology, Rheumatology, Endocrinology and Metabolism, Graduate School of Medicine, Dentistry and Pharmaceutical Sciences, Okayama University, 2-5-1 Shikata-cho, Okayama 700-8558, Japan; 2 Department of Nephrology, Aoe Clinic, 5-1-3, Aoe, Okayama 700-0941, Japan

**Keywords:** kidney organoid, stem cell, disease modeling, drug toxicity, drug screening, regenerative medicine

## Abstract

Kidney organoids, derived from stem cells, including pluripotent stem cells and adult progenitor cells, have been reported as three-dimensional in vitro models that reflect key aspects of kidney development, structure, and function. Advances in differentiation protocols and tissue engineering have enabled the generation of organoids that exhibit nephron-like structures, including glomerular and tubular structures. Kidney organoids have been widely applied in several directions, including disease modeling and therapeutic screening, drug nephrotoxicity evaluation, and regenerative medicine. In particular, kidney organoids offer a promising platform for studying genetic kidney diseases, such as polycystic kidney disease and congenital anomalies of the kidney and urinary tract (CAKUT), by allowing patient-specific modeling for the analysis of pathophysiology and therapeutic screening. Despite several current limitations, such as incomplete maturation, lack of full nephron segmentation, and variability between protocols and cell conditions, further technological innovations such as microfluidics and bioengineering may refine kidney organoid systems. This review highlights recent advances in kidney organoid research, outlines major applications, and discusses future directions to enhance their physiological relevance, functional maturity, and translational integration into preclinical and clinical nephrology.

## 1. Introduction

**Figure 1:**
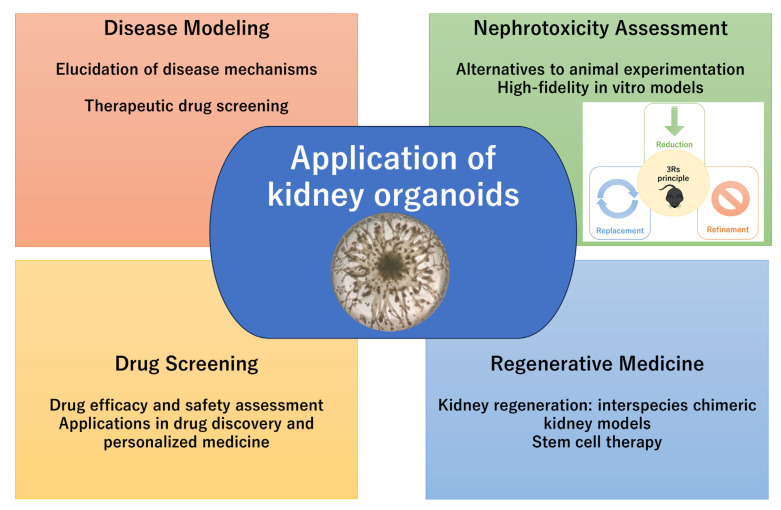
Application of kidney organoids.

The kidney is a structurally and functionally complex organ responsible for maintaining fluid and electrolyte balance, excreting waste products, and regulating hormones. Studying kidney development, disease, and drug-induced injury has historically relied on the combination of animal models and in vitro cell lines, both of which have significant limitations in mimicking the intricacy of human kidney physiology. The recent advent of three-dimensional (3D) kidney organoid technology has significantly advanced kidney research (Figure 1). Kidney organoids, primarily derived from pluripotent stem cells (PSCs), such as embryonic stem cells (ESCs) or induced pluripotent stem cells (iPSCs), are capable of self-organizing into nephron-like structures and recapitulating key aspects of early kidney development [1,2]. These organoids provide a promising platform to model human kidney diseases [3], investigate developmental pathways, and perform high-throughput drug screening in vitro [4].

Notably, kidney organoids have been shown to reflect key pathological features of kidney diseases, such as polycystic kidney disease (PKD) and nephrotic syndrome, as well as serving as predictive nephrotoxicity [5,6,7]. In addition, integration with bioengineering strategies (microfluidic chips and 3D bioprinting) may increase organoid maturation and scalability [8,9]. Despite several current limitations, including a lack of vascularization, incomplete nephron differentiation, and variability between batches, further technological innovations, such as microfluidics, bioengineering, and single-cell transcriptomics, may refine kidney organoid systems [10,11]. This review summarizes the recent advances in kidney organoid research, exploring their practical applications, and highlights future directions to enhance their physiological relevance and translational potential.

## 2. Development of Kidney Organoids

### 2.1. Overview of Kidney Developmental Biology

The development of the kidney is a highly organized and complex process that culminates in the formation of the metanephros. This process involves complex interactions between two key embryonic cells, the ureteric bud (UB) and the metanephric mesenchyme (MM). These tissues originate from the intermediate mesoderm and engage in reciprocal inductive signaling, which is essential for proper kidney morphogenesis [12,13,14]. The UB arises from the posterior portion of the Wolffian duct, invades into the MM through the regulation by the glial cell line-derived neurotrophic factor (GDNF) secreted by the MM and its receptor RET on the UB [15,16]. As the UB undergoes repetitive branching, it forms the collecting ducts. The MM contains nephron progenitor cells (NPCs) that are maintained in an undifferentiated state by transcription factors such as SIX2, WT1, PAX2, and OSR1 [17]. Under the induction of UB-derived signals, notably WNT9b and FGF, a subset of NPCs undergoes mesenchymal-to-epithelial transition (MET) and differentiates into the epithelial components of the nephron, including glomerulus, proximal tubule, loop of Henle, and distal tubule [18,19,20].

A variety of signaling pathways orchestrate this developmental process. WNT signaling (WNT4, WNT9b) is essential for MET and nephron induction [18]. BMP7 supports MM survival and proliferation [21], while FGF signaling (particularly FGF8 and FGF9) promotes cell differentiation and UB branching [22,23]. Notch signaling contributes to nephron segmentation and fate specification [24]. The temporal and spatial integration of these pathways regulates the formation of nephrons.

In humans, the metanephric kidney is unique among mammalian organs because nephron formation stops around birth and shows no regenerative capacity in adulthood. This developmental limitation underscores the importance of understanding nephrogenesis, both for regenerative medicine and disease modeling [25]. Insights from metanephric development have directly informed protocols for generating kidney organoids from PSCs. By mimicking the sequential activation of developmental cues, such as intermediate mesoderm induction, MM patterning, and MET, several groups have successfully created nephron-like structures in vitro [1,2]. These organoids exhibit nephron segments and can be used to model genetic kidney diseases, assess nephrotoxicity, and explore developmental defects [3]. However, achieving full structural and functional maturation still has several challenges, requiring further optimization of differentiation conditions and tissue engineering strategies [10].

### 2.2. Protocol for Differentiation of iPSC/ES Cells

The differentiation of human iPSCs or ESCs into kidney organoids is a multi-step protocol that aims to reproduce key stages of embryonic kidney development. This in vitro strategy typically mirrors the sequential induction of the primitive streak, intermediate mesoderm, and MM [2,26,27]. Despite protocol-specific differences, these methods generally follow a common developmental pathway, involving the sequential induction of the primitive streak, intermediate mesoderm, and MM, resulting in nephron formation. Takasato et al. demonstrated that transient activation of canonical WNT signaling with the GSK3β inhibitor CHIR99021 drives PSCs toward a posterior primitive streak identity, which can subsequently be patterned into intermediate mesoderm with fibroblast growth factor 9 (FGF9) and, in some protocols, bone morphogenetic protein 7 (BMP7) [1,28]. After several days of mesodermal induction, cells are aggregated into 3D spheroids or transferred to low-adhesion culture conditions to promote tissue organization. Continued exposure to FGF9 supports the maintenance and expansion of nephron progenitor populations within the developing MM, ultimately leading to the nephron-like structures through MET. Taguchi et al. established an alternative approach, guided by insights from mouse embryology, in which stage-specific signaling cues were used to generate nephron progenitors closely resembling in vivo counterparts [27]. Morizane et al. reported a protocol that emphasized the generation of a more homogeneous nephron progenitor population [2]. By optimizing growth factor exposure and culture conditions, this method reduced variability and the emergence of off-target cell types, thereby improving the reproducibility of organoid differentiation. Although these protocols differ in technical details, such as the concentrations of CHIR99021, the inclusion of BMP7, or the timing of aggregation, they share the same concept, including a stepwise recapitulation of embryonic kidney development in vitro. These studies established the foundation for kidney organoid research for applications in disease modeling, nephrotoxicity testing, and regenerative medicine. In addition to PSC-based protocols, our group established kidney organoids derived from adult rat kidney stem (rKS) cells [29]. Unlike PSC-based methods that require sequential recapitulation of early embryonic developmental stages, the rKS cell-based approach applies tissue-resident progenitors already committed to renal lineages. By supplementing the culture medium with a defined cocktail of five growth factors, including BMP7, FGF2, VEGF, EGF, and HGF, rKS cells can self-organize into 3D tubular organoids within 14–21 days. This simple protocol reproducibly generates structures that exhibit tubular morphology and express characteristic nephron markers, without the need for extended differentiation procedures.

Under these protocols, differentiating kidney organoids develop visible structures with distinct nephron-like segments, including glomeruli (expressing markers such as NPHS1 and PODXL), proximal tubules (LTL+), and distal segments [2,28]. However, full maturation, including vascularization and complex urine-concentrating function, remains limited in these standard in vitro protocols. To address these limitations, various refinements have been introduced. For example, dynamic culture systems such as spinning bioreactors or organ-on-a-chip platforms improve nutrient delivery and enhance maturation [8,9]. Vascularization can be induced through co-culture with endothelial cells or by transplanting the organoids into immunodeficient mice, where host-derived vasculature integrates with the developing tissue [30,31]. Recent advances in single-cell RNA sequencing and spatial transcriptomics are being applied to the refinement of these differentiation protocols [10,11].

### 2.3. Cell Type Characteristics in the Kidney Organoids

Kidney organoids generated from PSCs reproduce many of the key structural and cellular features of the developing human kidney. These organoids self-organize into 3D architectures that contain multiple renal cell types, mimicking the spatial organization and complexity of nephron units to a significant degree [1,2]. The glomerular-like structures in kidney organoids typically consist of podocyte-like cells expressing characteristic markers such as WT1, NPHS1 (nephrin), and PODXL (podocalyxin). These cells often form capillary loop-like configurations, although vascularization and filtration barrier function remain limited without in vivo transplantation or co-culture with endothelial cells [31]. Proximal tubules in organoids can be identified by brush border morphology and the expression of markers such as LTL (lotus tetragonolobus lectin), SLC3A1, and CUBN. These structures often demonstrate some functional uptake capabilities, such as albumin endocytosis [3]. Distal tubule-like cells express markers including ECAD (E-cadherin) and SLC12A1. Segmental patterning and polarity can be observed under appropriate culture conditions [11]. Interstitial cells are also present in kidney organoids. These include FOXD1+ stromal cells and PDGFRβ+ mesenchymal cells [32]. In some protocols, off-target cell types such as neurons or myocytes may arise, reflecting incomplete lineage specification [1]. Endothelial cells may be observed within organoids, but are typically sparse and not well-organized. Attempts to improve vascular integration have included co-culturing with iPSC-derived endothelial progenitors or transplanting organoids into mice, where host vasculature can infiltrate the tissue and enhance maturation [30].

While kidney organoids recapitulate many nephron components, including glomerular and tubular segments and surrounding stroma, the extent of their structural and functional fidelity remains limited. Advances in bioengineering, culture optimization, and spatial transcriptomic profiling may refine the identity and organization of renal cell types within these in vitro systems [10].

## 3. Technological Advances in Kidney Organoid Generation

### 3.1. Genome Editing with CRISPR/Cas9

Genome editing technologies, particularly the CRISPR/Cas9 system, have revolutionized kidney organoid research by enabling precise genetic manipulation of human PSCs. This technology facilitates both the modeling of genetic kidney diseases and the functional dissection of gene regulatory networks involved in nephrogenesis [7,33]. CRISPR/Cas9 uses a guide RNA to direct Cas9 to a specific genomic locus, where it creates a double-stranded break. Repair by non-homologous end joining (NHEJ) produces knockout mutations, while homology-directed repair (HDR) enables precise edits. The resulting gene-edited hPSCs can be differentiated into kidney organoids to investigate the effects of specific mutations [3]. One major application of CRISPR/Cas9 in kidney organoids is disease modeling, such as autosomal dominant polycystic kidney disease (ADPKD), nephrotic syndrome, and Alport syndrome. Despite its power, CRISPR editing in hPSCs requires careful validation due to risks of off-target effects, clonal variability, and potential differentiation biases. Clonal screening, sequencing, and functional assays are essential for confirming the fidelity and relevance of generated models.

### 3.2. Three-Dimensional Bioprinting and Microfluidics

The integration of kidney organoid technology with advanced bioengineering platforms, such as microfluidics, advanced the structural complexity, reproducibility, and physiological relevance of in vitro kidney models. The aim of these technologies is to cover key limitations of traditional organoid cultures, including variability in structure, insufficient vascularization, and limited functional maturation [34]. Three-dimensional bioprinting allows for the spatially controlled deposition of cells and biomaterials in defined patterns, which enable the construction of kidney tissue with improved organization. Using bioinks that compose extracellular matrix components and kidney progenitor cells, researchers may print structures that mimic nephron alignment, branching ureteric bud structures, or even vascular networks. This approach not only enhances the reproducibility of organoid formation but also allows for scale-up and standardization, critical for high-throughput drug screening [9]. Microfluidic devices, which are also known as organ-on-a-chip systems, may provide dynamic perfusion and mechanical stress that better reproduce the kidney’s physiological environment. In kidney-on-a-chip models, organoids are cultured in microchambers through which media can flow continuously, simulating blood perfusion and shear stress. This improves oxygen and nutrient delivery, facilitates waste removal, and promotes tissue maturation, particularly in tubular and endothelial compartments [35,36]. Furthermore, microfluidic systems allow for precise control of drug exposure and enable real-time monitoring of cellular responses [8,35,37,38].

### 3.3. Co-Culture System

Co-culture systems incorporating non-renal cell types such as endothelial cells and immune cells are applied to enhance the physiological relevance of kidney organoids. These approaches aim to reflect the multicellular microenvironment of the kidneys, where the immune system and vascularization play critical roles in both homeostasis and pathology. The integration of endothelial cells into organoid cultures may address the lack of vasculature, a key limitation of standard kidney organoids. Co-culture with human umbilical vein endothelial cells (HUVECs) or iPSC-derived endothelial progenitors has been shown to promote the formation of vascular-like networks within organoids [38,39]. When combined with microfluidic platforms or in vivo transplantation, these endothelial cells can further support perfusion and organoid maturation, particularly within glomerular and tubular structures [38]. Immune cell co-culture may also serve as a potential disease model. The incorporation of macrophages or T cells enables the study of immune-kidney interactions, which is relevant to glomerular and tubulointerstitial nephritis. A recent study demonstrated that infiltration of allogeneic T cells and macrophages into kidney organoids upon co-culture with PBMCs induced fibrosis-related changes and differential gene expression in stromal and epithelial compartments [40]. Furthermore, co-culture with monocytes/macrophages enhances the survival and differentiation of iPSCs into renal lineages via autophagy-mediated mechanisms [41].

### 3.4. High Throughput and Automation

Automation and high-throughput strategies are promising applications for the transformation of the kidney organoid system toward industrial-scale applications. These developments enable efficient screening pipelines for nephrotoxicity, disease modeling, and precision medicine with reducing labor, cost, and variability. Scale-up of kidney organoids with consistency and reproducibility is a major goal in translational research and drug discovery. Recent studies have shown the use of high-throughput and automated platforms in kidney organoid protocols. Using culture systems in 96- and 384-well plates, the parallel generation of hundreds of organoids under standardized conditions is possible. This approach significantly reduces reagent costs and enables systematic screening of nephrotoxic compounds or therapeutic candidates [3]. Automated liquid handling systems are also used to precisely control cell seeding, medium changes, and reagent addition, increasing reproducibility across batches. High-content imaging systems and machine learning-based image analysis tools are integrated to quantify organoid morphology, marker expression, and viability in an unbiased and scalable manner. These systems allow for the detection of subtle phenotypic changes in response to drug treatment or genetic modification [3]. Microfabricated devices, including microwell arrays and droplet-based platforms, further improve uniformity in organoid formation and facilitate real-time monitoring. Some platforms incorporate biosensors or microfluidics to provide dynamic readouts of function, such as barrier integrity or metabolic activity [42,43,44].

## 4. Applications of Kidney Organoids

Since the advent of iPSC-derived kidney organoids [1,2,27], a wide range of organoid models has been developed to investigate human kidney development, elucidate disease mechanisms, and advance therapeutic drug screening. For example, as a model of human kidney disease, it has been reported that exposing kidney organoids to hyperglycemic conditions induces characteristic features of diabetic kidney disease (DKD) [45]. Moreover, genetic disease models have been generated either by introducing mutations into healthy iPSCs using CRISPR/Cas9 or by reprogramming patient-derived somatic cells into iPSCs, resulting in organoids that display structural and functional abnormalities characteristic of the underlying disorder. Due to their multicellular complexity and ability to reflect human nephron structure, organoids provide a superior model compared to traditional monolayer cultures, particularly when assessing disease-specific responses [46]. Kidney organoids hold great potential in contexts where conventional animal models are difficult to reproduce human-specific pathophysiology, for example, lethal kidney diseases [47]. Moreover, the use of kidney organoids for in vitro drug screening has emerged as an important approach for evaluating the efficacy of candidate therapeutics targeting kidney diseases. The automation of kidney organoid culture and image analysis facilitated the establishment of a high-throughput screening platform [3]. This enables efficient screening of large compound libraries under standardized conditions.

Although these studies highlight the potential of kidney organoids as disease reflecting models, fully established systems have yet to be achieved. This is mainly due to the absence of essential in vivo components such as vasculature, blood flow, and immune cell infiltration. In addition, protocols to model long-term chronic stress, hypoxia, and metabolic dysregulation remain limited, underscoring the need for further refinement of organoid-based disease models. Various organoid-based disease models have been reported, demonstrating specific pathological features.

### 4.1. Organoids in Genetic and Congenital Kidney Diseases

Human PSCs-derived kidney organoids have enabled patient-specific modeling of numerous inherited and congenital kidney diseases. Compared with conventional two-dimensional or animal models, these systems capture species-specific molecular pathology and genetic diversity, thereby improving mechanistic understanding and supporting preclinical testing of therapeutic strategies (Table 1).

Alport syndrome is a hereditary nephropathy caused by mutations in the COL4A3, COL4A4, or COL4A5 genes encoding type IV collagen, leading to structural defects of the glomerular basement membrane (GBM) and progressive kidney dysfunction [48]. Although gene therapy, including recombinant adeno-associated virus–mediated delivery, gene editing, and antisense oligonucleotides, is under investigation, definitive curative treatments for Alport syndrome have not yet been implemented in clinical practice [49]. Using patient-derived iPSCs, kidney organoid models of Alport syndrome have recently been established. Kidney organoids derived from COL4A5-mutant iPSCs recapitulated the absence of the mature α3/α4/α5(IV) collagen network in the GBM, successfully modeling phenotype differences between mild and severe Alport syndrome [50]. Treatment with the chemical chaperone 4-phenylbutyric acid partially restored GBM abnormalities in mild Alport syndrome organoids, whereas an exon-skipping strategy using antisense oligonucleotides restored expression of the C-terminal region of COL4A5 in vitro [51]. While these models achieve high molecular and structural fidelity, the absence of vascularization and long-term filtration limits functional evaluation of progressive renal dysfunction.

Congenital nephrotic syndrome is a rare, inherited kidney disorder most commonly caused by mutations in NPHS1, NPHS2, WT1, LAMB2, or other podocyte-related genes, and is characterized by massive proteinuria, hypoalbuminemia, edema, and rapid progression to kidney dysfunction [52]. Patient-derived iPSCs with mutations in NPHS1 [53,54], NPHS2 [55,56], and WT1 [57] have been used to generate kidney organoids, enabling the recapitulation of disease phenotypes in vitro. However, because kidney organoids lack a functional filtration barrier connected to a urinary outflow system, direct assessment of albumin leakage (proteinuria) is not feasible. To overcome this limitation, the glomerular filtration barrier, which is responsible for blood filtration and the prevention of protein loss, has been recapitulated by seeding human podocytes and glomerular endothelial cells into microfluidic chips [58]. This approach is therefore expected to provide a promising platform for the evaluation of albumin permeability.

One of the well-established applications of kidney organoids is the modeling of ADPKD. ADPKD represents the most common genetic cause of CKD, accounting for up to 10% of end-stage renal diseases [59,60]. Freedman et al. generated ADPKD organoids from patient-derived or CRISPR/Cas9-edited iPSCs, demonstrating cyst-like structures [7]. Organoids have also been generated from iPSCs derived from ADPKD patients, and ADPKD models have been utilized to investigate disease mechanisms and to explore potential therapeutic agents [61,62,63,64,65,66,67,68]. Organoids derived from PKD1 or PKD2-mutant PSCs form fluid-filled cysts in vitro, closely resembling the cystogenesis observed in patient kidneys. Cystogenesis is strongly influenced by the extracellular microenvironment, where non-adherent culture conditions and cyclic AMP stimulation significantly enhanced cyst expansion, underscoring the importance of biomechanical and biochemical cues in disease modeling [5]. Moreover, microfluidic organoid-on-a-chip systems demonstrated that luminal glucose absorption and fluid shear stress drive cyst expansion, providing insights into biomechanical contributions to disease progression [69]. Large-scale live imaging technology was applied to screen 247 protein kinase inhibitors in ADPKD models, successfully identifying compounds that suppressed cyst expansion without impairing overall organoid growth, thereby demonstrating the feasibility of unbiased drug screening in this setting [70]. Moreover, long-term expandable nephron progenitor cell generates mature kidney organoids and, when applied to ADPKD, provide a platform for large-scale genetic editing and screening [71]. While ADPKD mouse models have been valuable for elucidating disease mechanisms and evaluating therapeutic strategies, they still have several limitations. These include the rapid disease progression compared with the slow course in humans, the embryonic lethality of complete gene knockouts, variability in disease severity across different models, and species-specific differences in therapeutic responses, all of which prevent them from fully reflecting the human condition [72]. By contrast, organoid-based systems make it possible to study ADPKD within the human genetic context. In addition, patient-derived organoid biobanks that cover a wide range of PKD1 and PKD2 mutations could support precision medicine approaches, helping to predict treatment responses and reveal genotype-specific disease mechanisms. Collectively, because of their robust phenotype and responsiveness to pharmacological interventions, ADPKD organoids currently represent one of the most functionally validated models for drug discovery and precision therapy.

Autosomal dominant tubulointerstitial kidney disease (ADTKD) is a rare inherited disorder characterized by an autosomal dominant inheritance pattern and progressive tubulointerstitial injury, leading to CKD [73]. ADTKD is increasingly recognized as one of the more common genetic causes of CKD by pathogenic variants in UMOD, MUC1, REN, HNF1B, and SEC61A1 [74,75]. These kidney organoid models have been generated for some of these subtypes, facilitating research into their molecular mechanisms and potential therapeutic strategies. ADTKD-uromodulin (ADTKD-UMOD) is caused by UMOD mutations, which have been identified in exons 3, 4, 5, and 7, with more than 100 pathogenic variants reported to date, most commonly missense mutations, but also including deletion and indel variants [76]. Kidney organoids differentiated from iPSCs derived from patients with ADTKD-UMOD were generated, and it was demonstrated that mutant UMOD abnormally accumulated within the cells, recapitulating the intracellular trafficking defect [77]. ADTKD-mucin1 (ADTKD-MUC1) is caused by a frameshift mutation in the GC-rich variable number of tandem repeats (VNTR) region of the MUC1 gene [78]. Using kidney organoids derived from iPSCs, the study established a disease model of Mucin 1 kidney disease. It demonstrated intracellular accumulation of an abnormal protein caused by a MUC1 frameshift mutation, which induced cellular stress responses [79]. Heterozygous Hepatocyte nuclear factor 1B (HNF1B) mutations are the monogenic cause of kidney dysplasia or ADTKD [80]. Kidney organoids derived from human ESCs and iPSCs successfully modeled heterozygous HNF1B-associated dysplastic kidney malformations, demonstrating structural abnormalities in both tubules and glomeruli, along with marked upregulation of GRIK3 (glutamate ionotropic receptor kainate type subunit 3) [81].

Juvenile nephronophthisis is an autosomal recessive kidney disorder characterized by progressive renal dysfunction in childhood and young adulthood, typically progressing to end-stage renal disease [82]. Using patient-derived and CRISPR-edited iPSCs, kidney organoids lacking NPHP1 exhibited frequent cyst formation under rotational suspension culture with downregulation of cilia-related genes, where this phenotype was rescued by NPHP1 overexpression [83].

Fabry disease is a rare X-linked inherited disorder that causes defects in the glycosphingolipid metabolic pathway that result from deficient or absent activity of the lysosomal enzyme α-galactosidase A (α-Gal A) [84]. Kidney organoids modeling Fabry disease have been generated from both patient-derived and CRISPR/Cas9-edited iPSCs with pathogenic GLA mutations. These organoids reflect the features of reduced α-galactosidase A activity, globotriaosylceramide (Gb3) accumulation with zebra bodies observed by transmission electron microscopy [85,86]. The rescue of the phenotype has been reported with enzyme replacement therapy and glutathione supplementation, and via gene-editing–based substrate reduction and CRISPR suppression of A4GALT (Gb3 synthase) [85,87]. Cystinosis is a rare, autosomal, recessive, lysosomal-storage disease caused by mutations in the CYSTINOSIN (CTNS) gene, encoding cystine transporter [88]. Hollywood et al. established human iPSCs-derived kidney organoids with CTNS deficiency that reflect key proximal tubular phenotypes of cystinosis, including cystine accumulation, lysosomal enlargement, defective autophagic flux, and increased apoptosis, and further identified combined cysteamine and mTOR inhibition as a potential therapeutic strategy [89]. Tuberous sclerosis complex (TSC) is an autosomal dominant disorder caused by mutations in TSC1 or TSC2, characterized in the kidney by angiomyolipoma (AML), renal cysts, and, less frequently, renal cell carcinoma, which together contribute to the risk of CKD [90]. Hernandez et al. generated kidney organoids from patient-derived iPSCs with TSC2 mutations, establishing a TSC2 homozygous knockout model. This model successfully reflects the pathological features of AML and cyst formation [91]. Pietrobon et al. used CRISPR/Cas9 gene editing to create human kidney organoids with homozygous knockout of either TSC1 or TSC2, and their study highlighted the essential roles of both genes in the development of renal manifestations associated with TSC [92].

In summary, ADPKD organoids currently provide the most robust and functionally validated platform for pharmacological testing, largely because cystogenesis offers a quantifiable and reproducible phenotype that can be modulated by biochemical and biomechanical cues. Similarly, Fabry disease organoids display clear metabolic and ultrastructural readouts, allowing direct assessment of therapeutic efficacy following enzyme replacement or gene editing. In contrast, Alport and congenital nephrotic syndrome organoids reproduce the molecular and structural defects of the glomerular basement membrane and slit diaphragm but remain limited by the absence of a perfused filtration barrier, preventing evaluation of albumin permeability or progressive functional decline. These differences highlight that the current depth of validation among genetic models is not only disease-dependent but also shaped by the nature of measurable functional outputs and the degree of vascular and metabolic maturation achievable in vitro. Bridging this gap will require integration of microfluidic perfusion, endothelial and immune co-culture, and long-term maturation systems to enable sustained filtration, matrix remodeling, and inflammatory crosstalk. Such approaches could transform structurally defined but functionally immature organoids into physiologically competent disease models capable of capturing chronic injury dynamics and therapeutic responsiveness. Collectively, these genetic and metabolic organoid systems exemplify the ongoing transition from descriptive morphology to mechanistic and functional precision, providing a scalable foundation for translational nephrology.

**Table 1:** Representative kidney organoid disease models and key features.

Disease	Genetics	Key Feature	Year	Ref.
Alport syndrome	COL4A5	Generation of a kidney organoid model of Alport syndrome The chemical chaperone (4-phenyl butyric acid)	2023	[50]
	COL4A5	Exon skipping	2024	[51]
Congenital nephrotic syndrome	NPHS1	Compound heterozygous NPHS1 mutations (affecting NEPHRIN and PODOCIN expression)	2018	[53]
	NPHS1	Slit diaphragm defects in the CNS kidney organoid model	2018	[54]
	NPHS2	Impaired NPHS2 expression, rescue by correction	2022	[55]
	NPHS2	Variant-specific podocin mislocalization	2023	[56]
	WT1	Delayed podocyte development; CRISPR rescue	2024	[57]
ADPKD	PKD1 PKD2	Generation of kidney organoid model of ADPKD	2015	[7]
	PKD1 PKD2	Highly efficient model of PKD cystogenesis	2017	[5]
	PKD1	ADPKD model using ureteric bud–derived organoids	2020	[61]
	PKD1	iPSC-derived kidney organoids from ADPKD patients	2020	[62]
	PKD1	Generation of kidney organoids from blood erythroid progenitor cells	2021	[63]
	PKD1	iPSC-derived organoids from ADPKD patients	2022	[64]
	PKD1 PKD2	Microfluidic organoid-on-a-chip systems	2022	[69]
	PKD1 PKD2	A scalable organoid model for high-throughput drug screening	2022	[70]
	PKD1	ADPKD model with collecting duct organoids Retinoic acid receptor agonists as therapeutic agents	2023	[65]
	PKD1 PKD2	Eukaryotic ribosomal selective glycosides as therapeutic agents	2024	[66]
	PKD1 PKD2	ADPKD model from expandable nephron progenitor cells	2024	[71]
	PKD1	Gene therapy using adenine base editing	2024	[67]
	PKD1	Recapitulation of early cyst formation and ciliary abnormalities	2025	[68]
ADTKD dysplastic kidney malformations (DKMs)	UMOD	Generation of kidney organoids from Patients with ADTKD-UMOD	2024	[77]
	MUC1	Trafficking defects and therapeutic agents in MUC1-mutant organoids	2019	[79]
	HNF1 HNF1B	Disease pathways elucidated in kidney organoid model	2024	[81]
Nephronophthisis	NPHP1	Cyst formation with downregulated cilia-related genes, rescued by NPHP1 complementation	2024	[83]
Fabry disease	GLA	Generation of Fabry disease organoid model α-Gal A supplementation and glutathione treatment	2021	[85]
	GLA	Recapitulation of disease phenotypes in organoids	2023	[86]
	GLA	Gene editing (A4GALT suppression)	2023	[87]
Cystinosis	CTNS	mTOR Inhibition Combination Therapy for Cystinosis	2020	[89]
Tuberous sclerosis complex (TSC)	TSC2;	TSC2^−^/^−^ kidney organoids model	2021	[91]
	TSC1; TSC2	Disease mechanism of TSC	2022	[92]

iPSCs: induced pluripotent stem cells; ADPKD: Autosomal Dominant Polycystic Kidney Disease; ADTKD: Autosomal Dominant Tubulointerstitial Kidney Disease; COL4A5: Collagen Type IV Alpha 5 Chain; NPHS1: Nephrin; NPHS2: Podocin; WT1: Wilms Tumor 1; PKD1: Polycystic Kidney Disease; UMOD: Uromodulin; MUC1: Mucin 1; HNF1: Hepatocyte Nuclear Factor 1; GLA: Galactosidase Alpha; CTNS: Cystinosin, Lysosomal Cystine Transporter; TSC2: Tuberous Sclerosis Complex.

### 4.2. Organoids for Modeling Acquired Kidney Injury and Fibrosis

Organoids have also been applied to model acquired and multifactorial kidney diseases, including diabetic kidney disease DKD and renal fibrosis. Compared with rodent models, human organoids provide species-specific transcriptional and metabolic profiles that more accurately reflect early human pathophysiology.

Rodent models of DKD are constrained by variability related to strain and genetic background, as well as by fundamental interspecies differences, thereby limiting their translational relevance to human disease [93]. To overcome these limitations, DKD has recently been modeled using human kidney organoids. Exposure of human kidney organoids to high oscillatory glucose leads to transcriptional changes, extracellular matrix alterations, and metabolic mitochondrial rewiring in tubular cells, which represent early hallmarks of kidney disease development induced by hyperglycemia [45]. Moreover, recent advances in organoid technology, including integration with organ-on-chip platforms, further enhance their potential for drug screening and precision medicine applications in DKD.

3D in vitro models of kidney fibrosis are indispensable for understanding its pathogenesis, progression, and therapeutic interventions [94,95]. Two-dimensional cell cultures present important limitations, including the absence of 3D tissue architecture and hierarchical organization, as well as significant differences in protein and gene expression, compared with 3D systems [96,97]. Recently, using human iPSC-derived kidney organoids, treatment with TGF-β1 has been shown to induce extracellular matrix production, reflecting pro-fibrotic signaling observed in chronic kidney disease (CKD) [98]. Kidney organoids are primarily composed of epithelial cell types and lack stromal populations, which imposes limitations on the assessment of fibrosis [99,100]. In addition, because most organoids lack interstitial fibroblasts and immune cell components, they do not fully capture the multicellular interactions involved in progressive fibrosis. Therefore, incorporation of stromal or immune cell populations and application of dynamic bioreactors may enhance matrix remodeling and inflammatory signaling, bridging the gap between acute injury models and chronic fibrotic pathologies [101].

Overall, organoid-based models of acquired injury provide valuable insights into early disease mechanisms and drug responses, but their translation to chronic, multicellular pathologies requires integration of vascular, stromal, and immune systems. Current kidney organoid systems remain largely avascular and immunologically immature, limiting their capacity to recapitulate chronic kidney disease features such as sustained inflammation, fibrosis, and progressive tissue remodeling. The absence of functional perfusion restricts long-term nutrient and oxygen delivery, leading to necrosis and loss of tissue polarity over time, while the lack of resident and infiltrating immune cells prevents modeling of immune-mediated injury, repair, and crosstalk with endothelial and mesenchymal compartments. To overcome these translational barriers, recent efforts have focused on co-culturing organoids with endothelial, macrophage, or lymphoid cell populations, as well as developing microfluidic platforms capable of continuous perfusion and immune cell trafficking, although these systems remain technically challenging and not yet standardized.

### 4.3. Organoids for Nephrotoxicity Testing and Drug Response Prediction

Preclinical nephrotoxicity testing remains a major challenge in drug development. Animal models often fail to predict human toxicity, and conventional 2D tubular cell lines lack complex nephron interactions as well as cell polarity. Kidney organoids, composed of diverse nephron segments derived from human PSCs, have become a valuable tool for preclinical nephrotoxicity assessment or investigation of drug-induced adverse effects. The kidney is particularly susceptible to toxic injury due to its role in drug metabolism and excretion [102]. Compared with conventional toxicity models, such as animal studies or two-dimensional proximal tubule cell lines, often lacking predictive power or translatability to human outcomes [103], kidney organoids provide a human-derived 3D system that is more likely to mimics enhanced cell–cell interactions, reproduction of diverse nephron segments, and a gene expression profile that more closely resembles the human kidney [104]. Moreover, the application of kidney organoids is also aligned with the principles of animal welfare, as the 3Rs (Replacement, Reduction, and Refinement) have been advocated and represent an urgent challenge [105]. Thus, research on kidney organoids for nephrotoxicity assessment is expected to serve as a promising in vitro alternative to animal models. Furthermore, the integration of iPSC-derived kidney organoids with organ-on-a-chip technology has emerged as a promising approach for nephrotoxicity assessment. Conventional organoid cultures lack vascularization and physiological perfusion, limiting their maturation and the accurate modeling of drug-induced injury [1]. By incorporating kidney organoids into microfluidic chip platforms, dynamic flow and shear stress can be applied, leading to improved tubular organization, enhanced metabolic activity, and more physiologically relevant responses [8,106]. At present, iPSC-derived kidney organoids provide a platform for segment-specific investigations of toxic injury.

For tubular injury assessment, application of tubular toxicants such as cisplatin and gentamicin to kidney organoids induced the upregulation of injury markers, including KIM-1, DNA damage marker, and apoptotic markers, demonstrating that organoids can recapitulate clinically relevant patterns of tubular injury [1,2]. Based on this information, kidney organoids have been explored as a tool for high-throughput drug screening, as they enable large-scale compound testing under well-controlled conditions. Advances such as standardized differentiation protocols, automated culture and imaging, and multidimensional phenotypic profiling have made these applications increasingly feasible [3,107,108]. Using our kidney tissue stem (KS) cell-derived organoid model, we histopathologically recapitulated cisplatin-induced tubular injury and confirmed robust induction of the DNA-damage marker γ-H2AX together with a reduction in the mitotic marker phospho-histone H3 [109]. We further employed the KS organoids to reproduce acute tubular injury associated with ingestion of a red yeast rice supplement (Beni-koji CholesteHelp), which became a major public health concern in Japan [110]. Treatment of KS-derived organoids with extracts from the implicated supplement batches caused tubular epithelial thinning, structural disruption, and cleaved caspase-3-positive apoptosis, closely mirroring the pathological findings in human biopsy specimens. These results provided the first experimental evidence demonstrating a causal link between supplement-derived toxicants and direct tubular injury, thereby establishing a proof-of-concept that organoid platforms can bridge experimental toxicology and clinical nephrology. Owing to their rapid differentiation, high reproducibility, and structural fidelity, KS cell-derived kidney organoids represent a practical and scalable platform for nephrotoxicity evaluation. Such systems may enable batch-specific safety testing of nutraceuticals and pharmaceuticals and facilitate translational nephrotoxicity assessment under controlled laboratory conditions, contributing to early detection of hazardous compounds and prevention of future supplement-related AKI incidents.

In addition to the tubular injury, glomerular injury has been modeled using organoid-derived podocytes. Several groups have demonstrated that kidney organoids can serve as platforms to model and evaluate glomerular injury. Kidney organoids generated from human iPSCs were shown to develop glomerular structures containing podocytes, as evidenced by the expression of markers such as NEPHRIN and PODXL [1,7]. Hale et al. demonstrated that organoid-derived glomeruli are suitable for toxicity screening using doxorubicin, highlighting their potential as an accessible approach for screening podocyte injury [53]. However, due to the absence of blood flow, these kidney organoids fail to recapitulate capillary loop formation and filtration barrier function as observed in vivo. To overcome this limitation, subcapsular transplantation of kidney organoids has been reported to induce vascularization and the formation of more mature glomeruli [30]. Using the organ-on-a-chip model, doxorubicin perfusion induced endothelial delamination and podocyte injury, thereby recapitulating drug-induced glomerular damage in vitro [111]. Further improvements in the detection of podocyte injury are anticipated through the application of organ-on-a-chip devices and co-culture with endothelial cells [30].

In addition to nephrotoxicity testing, kidney organoids have also been applied as promising tools for drug response prediction and precision nephrology. Because organoids can retain patient-specific genetic and epigenetic backgrounds, they enable the assessment of interindividual variability in drug efficacy and toxicity, providing a preclinical platform for personalized medicine. For instance, PSCs-derived organoids from patients with ADPKD have been used to evaluate the response to vasopressin V2 receptor antagonists, mTOR inhibitors, and retinoic acid receptor, recapitulating genotype-dependent drug sensitivity in vitro [65,112]. Integration with multi-omics and artificial intelligence (AI)-based image analysis may further enable high-throughput screening for drug responsiveness and biomarker discovery, bridging the gap between molecular profiling and therapeutic outcomes. Ultimately, such organoid-based pharmacological testing frameworks could guide patient stratification, dose optimization, and individualized therapeutic decision-making in nephrology.

Nevertheless, both genetic and nephrotoxic injury models share common challenges, including incomplete maturation, lack of perfusion, and absence of immune and vascular interfaces. In addition, current kidney organoids exhibit limited expression and functional diversity of renal drug transporters, including OAT1/3, OCT2, and MATE1, which restrict the accurate prediction of compound uptake, metabolism, and clearance. The lack of immune cell populations further hinders the modeling of inflammation-mediated injury and repair pathways that critically influence drug response and toxicity. Addressing these limitations through standardized functional benchmarks, flow-integrated co-culture systems, and cross-organ coupling with liver- or vascular-on-chip platforms will be essential for the reliable application of organoids in predictive pharmacology and toxicology.

### 4.4. Regenerative Medicine

In the field of regenerative medicine, PSCs-derived kidney organoids have emerged as a promising tool that bridges basic research and therapeutic applications. Kidney organoids may offer a novel approach to restore or replace damaged renal tissue. While fully functional transplantation-ready kidneys remain a long-term goal, organoids derived from human PSCs provide a critical intermediary step toward cell-based therapies and tissue engineering. Recently, renal subcapsular transplantation of kidney organoids has demonstrated the potential for vascular integration, glomerular maturation, and even limited urine filtration [8,30]. More recently, strategies to generate chimeric kidneys have been explored. Progenitor cell integration approaches for chimeric kidney generation have been reported [113], and the production of human-pig chimeric renal organoids has also been reported [114]. While significant hurdles remain, including immune compatibility, vascularization, and scalability, the use of kidney organoids may lead to future regenerative nephrology therapies.

Although significant hurdles remain, including immune compatibility, vascularization, and large-scale production, these strategies collectively represent the emerging frontier of organoid-based regenerative medicine, as well as disease modeling, drug screening, and toxicity assessment (Table 2). Future translation will require standardized manufacturing, ethical oversight, and long-term safety assessment to realize their full potential as cell-based therapies.

## 5. Limitations and Challenges

Although kidney organoid research has advanced considerably, important limitations and technical barriers remain, restricting its translation into clinical and industrial applications. These challenges include biological immaturity, technical variability, and regulatory constraints.

A major limitation of current kidney organoid systems is their incomplete structural and functional maturation. Organoids often showed underdeveloped glomeruli and immature tubular segments, reflecting incomplete activation of developmental signaling pathways, including WNT, BMP, and FGF axes. Insufficient morphogen gradient formation and the absence of mechanical cues, such as shear stress and hydrostatic pressure, further impair nephron patterning and segment-specific differentiation. Consequently, podocyte markers such as NPHS1 and WT1, as well as tubular transporters, including SLC22A2 and SLC12A1, remain at fetal-like expression levels compared to adult kidneys.

One critical challenge is the lack of perfused vascularization, which restricts nutrient and oxygen delivery and limits glomerular filtration barrier formation. In static culture, the hypoxic core of organoids leads to downregulation of endothelial markers (CD31, VE-cadherin) and increased necrosis. Although in vivo transplantation and bioengineering strategies have shown partial improvement, achieving adult-like renal function remains a key challenge [115]. By contrast, microfluidic perfusion systems and dynamic bioreactors have been shown to enhance endothelial network formation by approximately 3-fold and improve oxygenation relative to static conditions [8]. As a result, kidney organoids cultured under flow exhibited more mature glomerular and tubular compartments, improved epithelial polarity, and upregulated expression of adult kidney markers, including PODXL and LTL, compared with static conditions. Furthermore, perfused organoids demonstrated morphological features reminiscent of capillary loop formation during embryonic nephrogenesis, suggesting that biomechanical cues such as fluid shear stress contribute to vascular and epithelial maturation. Furthermore, ECM scaffolding and organoid-on-chip platforms incorporating extracellular matrix hydrogels (Matrigel, or decellularized kidney ECM) have been reported to improve nephron organization and mechanical stability, promoting the deposition of basement membrane components [116,117]. Although these bioengineering methods have collectively advanced the structural and vascular maturation of kidney organoids, achieving fully perfusable microvascular networks and adult-like renal function remains an ongoing challenge. Nevertheless, the ability to promote flow-based vascular development in vitro may provide promising foundations for simple and practical millifluidic chip-based culture systems that can support functional nephron development.

Current kidney organoid differentiation systems still have the problem of batch-to-batch variability. Factors contributing to inconsistency include iPSC line quality, culture conditions, and operator technique. Single-cell RNA sequencing studies have revealed significant variation in cell proportions and maturation states between different PSC lines and experiments, often attributed to off-target cell populations [10,118]. This variation inhibits reproducibility, complicates experimental interpretation, and limits scalability for drug screening. While high-throughput platforms have been developed to cover these issues, further improvements in protocol robustness and quality control remain required. In addition, long-term maintenance of kidney organoids is another important technical challenge, including increased risk of necrosis, loss of structural integrity, and cellular senescence. Most organoids can be maintained for 2–4 weeks. Long-term culture is essential for modeling CKD and for testing long-duration drug exposures. Developing perfusion systems, optimizing culture media, and incorporating supporting cell types such as vasculature or immune cells may help extend viability and function [43,119].

The future of the kidney organoids system aims to integrate into personalized medicine, supported by advances in AI, organoid biobanking, and clinical translation. AI-driven image analysis and data integration have the potential to overcome current limitations of variability and scalability. For example, machine learning algorithms enable high-throughput, automated quantification of organoid morphology, segmentation of organoid substructures, and objective assessment of maturation status, reduce operator bias, and facilitate standardization of differentiation protocols across laboratories [120,121,122]. In addition, multi-omics integration platforms combining transcriptomic, proteomic, and metabolomic datasets can identify molecular signatures associated with nephrotoxicity, fibrosis, or podocyte injury, thereby improving the predictive validity of organoid-based assays for drug safety testing and disease modeling [123,124,125]. In addition, establishing large-scale organoid biobanks from genetically diverse patient populations will allow genotype-phenotype correlation analyses and individualized drug screening. Such biobanks are already being initiated through national and international programs (e.g., the HCA: Organoid Atlas within the Human Cell Atlas), aiming to standardize metadata, quality metrics, and ethical governance. From a translational perspective, further progress will depend on the development of organoid platforms that meet toxicological validation, regulatory compliance, and GMP-grade manufacturing standards. Integration with microphysiological systems and organ-on-chip technologies may facilitate adoption in preclinical drug evaluation, particularly under evolving regulatory frameworks. Notably, the FDA Modernization Act 2.0, as well as Good In Vitro Method Practices (GIVIMP) and broader New Approach Methodologies (NAMs), support the use of advanced nonclinical models such as organoids and organs-on-chip as potential alternatives to animal studies in preclinical drug evaluation and regulatory assessment [126].

Finally, the use of human iPSCs, ESCs, and chimeric organoid models necessitates comprehensive and continuous bioethical reflection. Ethical considerations extend beyond conventional issues of consent and privacy to include questions of human–animal boundary, neural or reproductive potential, and the moral status of advanced organoid systems. In particular, the creation of human–animal chimeric organoids raise concerns regarding species identity and potential human cell contribution to germline or central nervous tissues [127]. Likewise, cerebral organoids that exhibit spontaneous electrophysiological activity demand discussion on the possibility of sentience and the need for refined oversight mechanisms [128]. To ensure responsible innovation, international bodies such as the ISSCR and OECD have developed guidelines that emphasize principles like transparency, accountability, reproducibility, and stakeholder engagement in stem cell and in vitro research.

Collectively, the integration of AI systems, standardized biobanking, and compliant bioengineering platforms will accelerate the transformation of kidney organoids from experimental tools into clinically relevant, patient-specific diagnostic and therapeutic models that may ultimately contribute to regenerative nephrology. Kidney organoids have the potential to reshape nephrology by enabling precise, patient-specific diagnostics, therapeutics, and tissue replacement strategies [129]. The use of human iPSCs and ESCs raises ethical and regulatory considerations, including consent, privacy, and appropriate governance. International harmonization will be essential for clinical translation [130].

### 5.1. Toward a Quantitative Framework for Kidney Organoid Validation

To advance kidney organoid research from descriptive observations toward measurable validation, we would propose three quantitative parameters: Maturity Index (MI), Reproducibility Index (RI), and Translational Potential Index (TPI). MI represents the ratio of adult-type to fetal-type marker expression or functional outcomes, including albumin uptake, transporter activity. RI may cover batch-to-batch variability and consistency. TPI integrates parameters, which reflect suitability for drug testing, disease modeling, and regulatory qualification. For example, comparative evaluation indicates that PSC-derived organoids exhibit higher MI, but lower RI due to protocol complexity. In contrast, adult KS-derived organoids may indicate higher RI and TPI for nephrotoxicity testing, because of the simplicity and structural stability. Integration of microfluidic perfusion and immune co-culture is expected to improve three parameters. These quantification and validation systems will support the establishment of quantitative benchmarks.

### 5.2. Future Roadmap

For the next decade, in the kidney organoid system to move experimental biology toward clinical translation, we propose a four-step translational roadmap that connects discovery to application through quantitative, multidisciplinary convergence.

Step 1: Functional Benchmarking

Building the proposed index (MI, RI, TPI) may establish quantitative validation metrics that define organoid quality and maturity across laboratories. These indices should be quantified by standardized assays assessing transporter activity, such as OAT1/3 and OCT2, and metabolic profiling. Multi-omics datasets, including transcriptome, proteome, and metabolome, may reproduce adult human kidney signatures, providing appropriate organoid validation.

Step 2: Flow and Immune Integration

Another important step toward physiological reproduction is the incorporation of dynamic perfusion and immune systems. Microfluidic perfusion systems can reproduce mechanical shear stress, oxygen gradients, and nutrient flow, which may support nephron maturation and vascular stabilization. Co-culture with endothelial cells and immune cells, such as macrophages and T cells, may reproduce functional interactions essential for modeling inflammation and regeneration. These integrations in the organoid system will serve as advanced models for CKD and drug-induced injury. Combining flow systems with 3D bioprinting may further enable scalable, spatially patterned nephron assembly compatible with organ-on-chip.

Step 3: AI-Driven Quality Control and Biobank Network

AI and machine learning systems may offer transformative solutions to overcome variability and subjectivity in organoid analysis. Automated imaging and deep-learning systems may quantify morphological and molecular parameters, providing unbiased assessments of differentiation efficiency, cell composition, and structural integrity. Integration with multi-omics databases and organoid biobanks will allow the construction of population-level reference maps that capture genetic diversity and disease heterogeneity. Interconnecting organoid biobanks through standardized metadata and interoperable digital platforms will facilitate cross-laboratory reproducibility, predictive modeling of drug response, and precision nephrology applications.

Step 4: Regulatory Application

The ultimate goal is the regulatory acceptance and clinical implementation of organoid-based systems. To achieve this, organoid platforms must align with international initiatives promoting NAMs, including the FDA Modernization Act 2.0. Defining performance standards, qualification criteria, and reporting templates for organoid-based assays will facilitate their use in toxicological evaluation and drug development pipelines. In parallel, ethical and data-governance systems are required to ensure the responsible use of human-derived cells and biobank data. Transparent oversight and cross-agency collaboration will accelerate the transition of organoid research from laboratory innovation to clinical translation.

Collectively, these four steps define a comprehensive translational platform that moves kidney organoids from descriptive research models toward validated and standardized platforms. By integrating biology, engineering, and ethics under quantifiable benchmarks, kidney organoids can evolve into core infrastructures for precision nephrology, enabling mechanistic disease modeling, individualized drug testing, and ultimately regenerative renal therapies.

## 6. Conclusions

Kidney organoids are progressing from descriptive 3D cultures toward quantifiable, regulatory-grade microphysiological systems. Realization of their full potential requires integrating biological advancement, engineering rigor, and ethical accountability within a unified, measurable framework. To facilitate this transition, quantitative performance metrics and harmonized documentation are required to bridge preclinical innovation with regulatory and clinical frameworks.

## Figures and Tables

**Table 2:** Comparative summary of kidney organoid applications.

Category	Representative Models	Validation Level	Advantage	Translational
Genetic Diseases	ADPKD, Alport, Fabry	Phenotype/Molecules/ Partial Functional	Enable analysis of disease mechanisms and drug screening	Moderate
Acquired Kidney Injury and Fibrosis	DKD Fibrosis	Molecular/ Partial functional	Model early metabolic stress and fibrotic signaling	Low
Toxicity assessment	Cisplatin, Gentamicin, Doxorubicin	Structural/Functional	Reflect human-relevant nephrotoxicity; suitable for mechanistic and high-throughput screening	Moderate
Regenerative medicine	Transplantation	Structural	Potential for renal replacement	Emerging

ADPKD: Autosomal Dominant Polycystic Kidney Disease; DKD: Diabetic Kidney Disease.

## Data Availability

There are no datasets generated during and/or analyzed during the current study.
